# Self-Cleaning Antibacterial Composite Coating of Fluorinated Acrylic Resin and Ag/SiO_2_ Nanoparticles with Quaternary Ammonium

**DOI:** 10.3390/polym16131885

**Published:** 2024-07-01

**Authors:** Jiangdong Gu, Qiufeng An, Meng-chen Huang, Ping Ge, Chao-hua Xue

**Affiliations:** 1 College of Chemistry and Chemical Engineering, Shaanxi University of Science and Technology, Xi’an 710021, China; bs200811016@sust.edu.cn (J.G.); g20950445@163.com (P.G.); 2 College of Bioresources Chemical and Materials Engineering, Shaanxi University of Science and Technology, Xi’an 710021, China; bs210111014@sust.edu.cn

**Keywords:** antibacterial coating, bactericidal effect, quaternary ammonium compounds, superhydrophobicity

## Abstract

With improvements in living standards, the demand for antibacterial self-cleaning coatings has significantly increased. In this work, self-cleaning coatings with antibacterial properties were fabricated by spray-coating a composite of fluorinated acrylic resin and Ag/SiO_2_ nanoparticles with quaternary ammonium salts. The synergistic action of the quaternary ammonium salts and silver nanostructures caused the coating to show a dual antibacterial effect. The Ag/SiO_2_ nanoparticles roughened the coating’s surface and, in combination with the fluorinated chains, provided the surface a superhydrophobic self-cleaning property with a contact angle of 156° and a sliding angle of less than 2°. Notably, the composite coating withstood 100 abrasion cycles without losing its superhydrophobicity and the contact angle is still exceeded 150° after 60 h of immersion solutions with different pH values, demonstrating outstanding wear resistance and acid/alkali stability. The incorporation of nanostructured antibacterial agents was effective in improving the roughness and antibacterial properties of the low-surface-energy resin, resulting in a self-cleaning antibacterial composite coating. This method may pave a new route for the design of functional coating materials with excellent overall performance.

## 1. Introduction

The human body is frequently exposed to large numbers of pathogenic bacteria, which pose a threat to human physical health. Specifically, the spread of bacteria on object surfaces is the leading cause of direct population infection [1,2,3]. Therefore, developing new and effective antimicrobial materials is imperative. Owing to their large surface area and good antimicrobial properties, antimicrobial coatings have been utilized in various fields, including medical devices, marine corrosion, and food processing. These coatings not only prevent biofilm formation and bacterial growth but also reduce the microbial load in critical environments [4,5,6]. Typically, coated materials with antimicrobial properties are produced by two primary strategies. One is to prevent bacteria from adhering to the material’s surface, and the other is to directly kill bacteria that come into contact with the surface. The most prevalent anti-adhesion strategy involves the preparation of superhydrophobic coatings with a static contact angle (CA) greater than 150° and a sliding angle (SA) less than 10° [7,8,9]. There are numerous superhydrophobic surfaces in nature, such as lotus leaves, insect wings, and spider feet. These superhydrophobic surfaces can be recreated by patterning their structures and passivating their coatings using low-surface-energy molecules. Various techniques, including photolithography, the sol–gel method, plasma etching, anodic oxidation, and chemical etching, have been used to prepare coating materials with superhydrophobicity [10,11,12,13]. These materials can be utilized in self-cleaning fabrics, corrosion protection, barrier reduction, and biofouling-resistant surfaces [14,15,16]. Superhydrophobic coating materials only effectively prevent bacterial adhesion for a limited time. The use of antibacterial agents to prepare superhydrophobic coatings could enhance the roughness and hydrophobicity of the coatings. In addition, the use of antibacterial agents could prevent biological contamination and eliminate bacteria to increase the durability of coatings. Typically, inorganic or organic antibacterial agents are used, and silver-based materials such as metallic silver, silver salts, and silver nanoparticles (Ag NPs) are the most effective and long-lasting options [17,18,19]. Among silver-based antimicrobial materials, silver nanoparticles have attracted much attention due to their antibacterial effects on various microorganisms and low toxicity to human cells. Meanwhile, the antibacterial properties of silver-based materials are closely related to their size, shape, and surface condition. Therefore, various Ag NPs are synthesized to create effective Ag NP-based antimicrobial nanomaterials, which are subsequently applied as antimicrobial coatings. However, the poor dispersion and facile sedimentation property of Ag NPs limit their practical application owing to their small size, large specific surface area, and abundance of surface-active sites. Consequently, inorganic substances such as SiO_2_ are typically used as carriers to encapsulate Ag NPs. Encapsulated Ag NPs are deposited on a substrate’s surface, thereby improving its roughness and consequently enhancing the substrate’s hydrophobicity. Meanwhile, the release rate of Ag^+^ could be slowed down owing to the protection of carriers, resulting in increased bactericidal efficiency [20]. Additionally, quaternary ammonium salts are the most widely used, cost-effective, and rapid-acting contact organic fungicides [21,22]. As an efficient type of surface modifier, quaternary ammonium compounds are frequently used to functionalize polymers or particles to boost their antimicrobial performance [23,24,25]. However, owing to their specific secretions and metabolites, bacteria inevitably adhere to the surfaces of materials containing positively charged quaternary ammonium and form biofilms, which are difficult to remove; therefore, their antimicrobial efficacy is significantly reduced. Combining a superhydrophobic coating with a biocide could maximize the deterrence of bacterial adhesion as well as kill and remove adhered bacteria. Therefore, combining superhydrophobic antifouling properties and fast, contact-killing surface antimicrobial activity has become desirable for the fabrication of durable antimicrobial coatings [26,27].

Herein, we fabricated superhydrophobic self-cleaning coatings with an antibacterial property by spray-coating composites of fluorinated acrylic resin and Ag/SiO_2_ nanoparticles with fluorinated chain and quaternary ammonium salts (Ag@FQS). The introduced Ag@FQS were employed to provide a micro–nanoscale rough coating structure, and the fluorinated long-chain silane and the fluorinated acrylic resin were intermixed well to enhance the wear resistance of the coating, making it possible to retain superhydrophobicity after repeated abrasion cycles. Notably, the synergistic effect of the quaternary ammonium salts and nanosilver provided a strong antimicrobial effect, and plate-counting and inhibition circle experiments revealed that the composite coating exhibited good anti-microbial adhesion, as well as antimicrobial functions.

## 2. Experimental

### 2.1. Materials and Reagents

4,4′-dicyclohexylmethane diisocyanate (HMDI) was produced by Shandong Jiaying Chemical Technology Co., Ltd. Hexafluoroisopropanol (HFIP) was purchased from Changzhou Lingda Chemicals Co., Ltd. Hydroxyethyl acrylate (HEAA) was obtained from Tianjin Kemio Chemical Reagent Co., Ltd., Tianjin, China. Octadecyl methacrylate (SMA) and perfluorohexyl ethyl acrylate (FAA) were provided by Shanghai Macklin Biochemical Reagent Co., Ltd., Shanghai, China. Ethyl acetate (EAC) and 4-methyl-2-pentanone (MIBK) were purchased from Tianjin Hedong Hongyan Reagent Factory. Dibenzoyl peroxide (BPO) was obtained from Taizhou Sea Chemical Industry Co., Ltd., Taizhou, China. *Staphylococcus aureus* (*S. aureus*) and *Escherichia coli* (*E. coli*) were obtained from IMCAS. Perfluorohexylethyltrimethoxysilane (FTMS) was purchased from Jinan Guochentaifu Chemicals Co., Ltd., Jinan, China. (Trimethoxy silpropyl) octadecyl dimethyl ammonium chloride (DC-5700) was purchased from the American Dow Corning corporation, Midland, MI, USA. Ethyl orthosilicate (TEOS) was purchased from Harbin Xuejia Fluorine Silicon Co., Ltd., Harbin, China. Silver nanoparticle powder (Ag NPs, 25 nm)was purchased from Beijing Deke Island Gold Technology Co., Ltd., Beijing, China. A hexamethylene–polyisocyanate curing agent (N-3390, Industrial grade) was purchased from Dongguan Haozhan Polymer Materials Co., Ltd., Dongguan, China.

### 2.2. Synthesis of Hydrophobic Fluorinated Acrylic Resin

**Figure 1:**
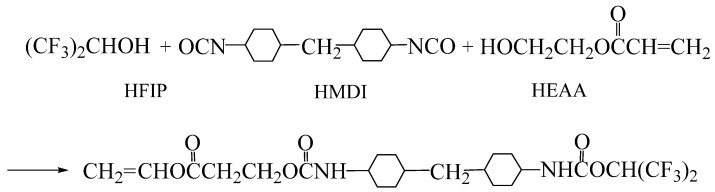
Synthesis of HFE.

First, 4-(hexafluoropropyloxy formamide) cyclohexyl-4′-(acryloxy ethoxy formamide) cyclohexyl methane, named HFE, which contained double bonds, was synthesized according to the reaction shown in Figure 1. Specifically, in a dry reactor equipped with a thermometer, reflux condenser, and electric stirrer, 7.87 g of 4,4′-dicyclohexylmethane diisocyanate (HMDI), 5.04 g of hexafluoroisopropanol (HFIP), and 10.92 g of a mixed solvent of ethyl acetate (EAC)/methyl isobutyl ketone (MIBK) with a mass ratio of 1:1 were added in turn and stirred for 60 min at 70 °C. Then 3.48 g of HEAA and a small amount of the EAC/MIBK solvent were added to ensure the total mass of the HMDI, HFIP, and HEAA accounted for 60 wt% of the mixture. Finally, the reaction was further carried out for 2 h to obtain polymerizable monomer HFE.

**Figure 2:**
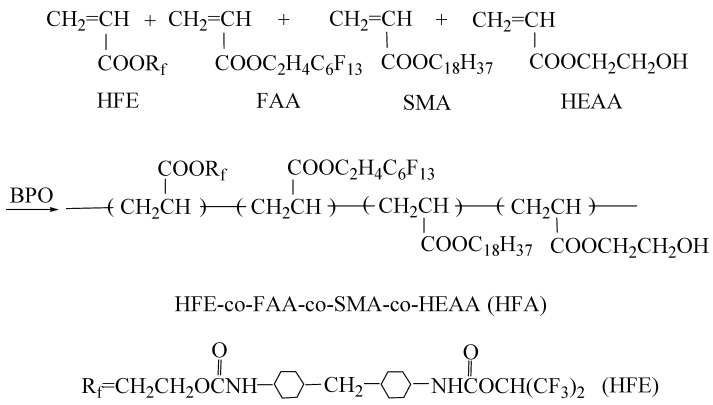
Route of HFA synthesis.

Secondly, 5.00 g of HEAA, 3.99 g of FAA, 9.98 g of SMA, and 0.34 g of BPO were added to the mixed EAC/MIBK solvent with 60 wt% content which was prepared after stirring (mixture A). Then, 1/3 of mixture A was poured into the above polymerizable monomer HFE and heated at 80 °C for 30 min while stirring. Finally, the other 2/3 of mixture A was added within 2 h, and the reaction was continued for 4 h to obtain a viscous, milky, and hydrophobic fluorinated acrylate resin referred to as HFA. The synthesis process is shown in Figure 2.

### 2.3. Preparation of Ag/SiO_2_ Nanoparticles with Fluorinated Chain and Quaternary Ammonium Salts

**Figure 3:**
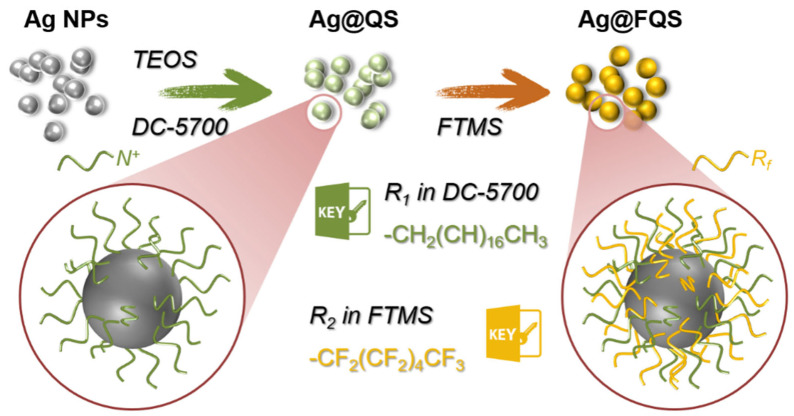
Schematic process for synthesis of Ag@FQS.

First, 0.5 g of silver powder and 10 g of absolute ethanol were mixed, ultrasonically dispersed for 30 min, and poured into a three-neck flask equipped with a thermometer, stirrer, and reflux condenser. Second, the mixture was heated to 80 °C, followed by the addition of 8.28 g of (trimethoxysilylpropyl) octadecyl dimethylammonium chloride (DC-5700) and 5.58 g of ethyl orthosilicate (TEOS). The pH was then adjusted to 4.0 using a diluted HCl solution, and 3.35 g of deionized water was slowly added and stirred for 1 h to form silver nanoparticles coated with quaternary ammonium salts (Ag@QS) (Figure 3). After that, 4.62 g of FTMS was diluted with ethanol to 50 wt% and added to the above mixture. Finally, the reaction was continued for 10 h to obtain Ag/SiO_2_ nanoparticles coated with fluorinated chains and quaternary ammonium salts (Ag@FQS) (Figure 3). Using the same method, pure SiO_2_ nanoparticles modified with fluorinated chains and quaternary ammonium salts were prepared via the hydrolytic co-condensation of TEOS, DC-5700, and FTMS without Ag NPs; they were named SiO_2_@FQS for a comparison study.

### 2.4. Preparation of Superhydrophobic Antibacterial Coating of HFA/Ag@FQS

**Figure 4:**
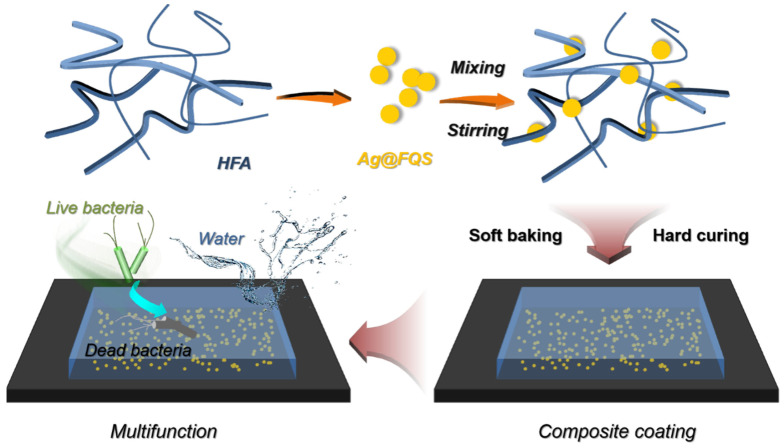
Fabrication of composite coating of HFA/Ag@FQS.

A 0.13 g amount of curing agent, N-3390, 4.00 g of HFA, and 0.22 g of Ag@FQS were mixed in 1.00 g of EAC and ultrasonically dispersed for 10 min. The mixture was cast on a clean glass sheet, and then cured at 120 °C for 0.5 h to obtain a superhydrophobic self-cleaning antibacterial coating of HFA/Ag@FQS (Figure 4). By controlling the weight percentage of the loaded bactericidal nanoparticles of Ag@FQS in the coating to be 3%, 6%, 9%, and 12%, composite coatings were produced and named HFA/Ag-3@FQS, HFA/Ag-6@FQS, HFA/Ag-9@FQS, and HFA/Ag-12@FQS.

### 2.5. Characterization

Fourier transform infrared spectroscopy (FTIR, Bruker Vector-22, Lubken, Germany) was used to detect the functional groups and chemical bonds of each substance. X-ray photoelectron spectroscopy (XPS, Kratos Axis Supra, Kyoto, Japan) was used to determine the composition, distribution, and valence states of the elements of the coating. X-ray single-crystal diffraction (XRD, Tongda TD-3500, Dandong, China) was used to detect the crystal shape of the samples, using a scanning area of 5~85°. The film morphology of each sample was characterized using a field-emission scanning electron microscope (FE-SEM, Thermo Scientific Verios-460L, Waltham, Massachusetts, America); prior to characterization, gold was sputtered on the test sample for 60 s under low-temperature vacuum conditions. A transmission electron microscope (TEM, FEI Tecnai G2 F20, Chicago, America) was used to characterize the internal structure of the film after it was ground. Atomic force probe scanning electron microscopy (AFM, Seiko, SPI-3800N/SPA-400, Chitose, Japan) was used to characterize the roughness of the coating surface. The water contact angle (CA) and sliding angle (SA) of the coating surface were determined on an optical-contact-angle-measuring device (OCA, Jinxiang JC2000C1, Shanghai, China). The deionized water droplet used to determine the CA was 8 μL, and for the SA, it was 10 μL. The values obtained at more than five different points on each sample were averaged as the values of the CA and SA. The mechanical stability of the samples was tested through an abrasion resistance test with 240-mesh sandpaper. When the as-prepared coating was in touch with the sandpaper, a horizontal external push (100 g) made the sample move forward 13 cm slowly along a ruler, which was designated one cycle. Then, the CA and SA were tested after each cycle. For a chemical stability evaluation, the coating was immersed in solutions with different pH levels for 60 h, followed by measuring the values of the CA and SA. To investigate the coating’s self-cleaning functionality, a specific amount of soil was placed on the superhydrophobic coating at a slightly inclined position, and water droplets were dropped on the soil to determine if the soil was removed by water droplets rolling away.

The preparation of Luria–Bertani (LB) liquid medium, the culture of Escherichia coli and Staphylococcus aureus, a plate count, and a bacteriostatic zone test were carried out. (1) For the preparation of the LB liquid medium, the method specified in GB/T 38483-2020 was used: 25 g of LB broth powder was first dissolved in 1000 mL of deionized water in an Erlenmeyer flask; then, the liquid was sterilized for 20 min under a high temperature in a sterilizing pot. After that, the flask was placed in a refrigerator at 4 °C for later use. (2) For the culture of *Escherichia coli* and *Staphylococcus aureus*, an inoculating loop was used to scrape colonies of *Escherichia coli* and *Staphylococcus aureus* on a sterile, ultra-clean worktable; then, the colonies were inoculated into the prepared LB liquid medium, sealed, and placed in a constant-temperature shaker at 37 °C for 8 h. (3) The determination of the antibacterial performance of the sample against *Staphylococcus aureus* (*S. aureus*) and *Escherichia coli* (*E. coli*) using the LB plate method was as follows: taking the number of colonies in the LB medium of *E. coli* as an example, 1 mL of the cultured *Escherichia coli* bacterial liquid was in full contact with the antibacterial material, incubated at 37 °C in an incubator for 24 h, and then removed and diluted with deionized water to a concentration of 10^9^ CFU/mL. At the same time, the diluted bacterial solution was inoculated on an LB plate on a sterile workbench coated evenly and placed in a 37 °C incubator for 8 h. The number of colonies on the LB plate and the dilution factor were observed to obtain the number of Escherichia coli present in the liquid, and the same method was used to calculate the number of bacteria in the Staphylococcus aureus liquid. (4) For the bacteriostatic zone test, 0.5 mL each of *Staphylococcus aureus* (*S. aureus*) and *Escherichia coli* (*E. coli*) at a concentration of 1×10^7^ CFU/mL was spread evenly on the LB liquid medium 3 times with a spreader, and 6 mm of paper spread with the sample was placed onto the LB medium after a solidification treatment. The LB medium was cultivated at a constant temperature of 37 °C for 24 h in a constant-temperature and constant-humidity incubator. The diameter of the bacteriostatic zone was measured using a vernier caliper.

## 3. Results and Discussion

### 3.1. Chemical Composition and Morphologies of HFA/Ag@FQS

**Figure 5:**
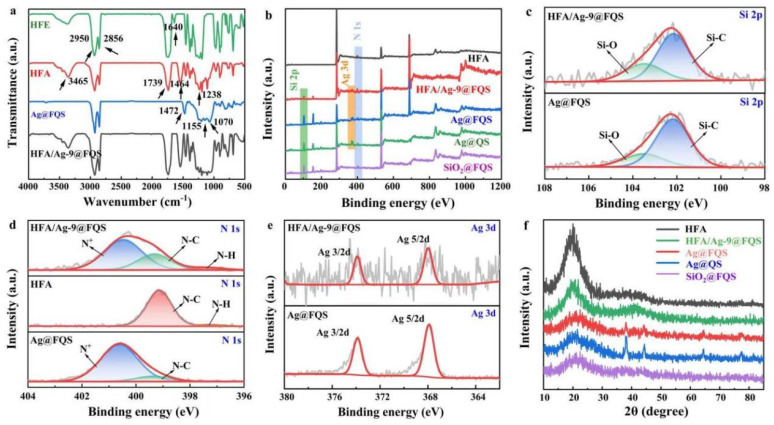
(**a**) FTIR spectra of HFE, HFA, Ag@FQS, and HFA/Ag@FQS. (**b**) XPS survey spectra of HFA, HFA/Ag-9@FQS, Ag@FQS, Ag@QS, and SiO_2_@FQS. XPS high-resolution spectra of (**c**) Si2p, (**d**) N1s, and (**e**) Ag3d. (**f**) XRD patterns of HFA, HFA/Ag-9@FQS, Ag@FQS, Ag@QS, and SiO_2_@FQS.

Figure 5a shows the FTIR spectra of the HFE, HFA, Ag@FQS, and HFA/Ag@FQS. The medium-strength peak at 3348 cm^−1^ is a N-H stretching vibration in a NHCOOH carbamic ester for HFE [28]. There is no characteristic absorption peak of -N=C=O at 2213 cm^−1^, indicating a complete reaction between the isocyanate group and the hydroxyl group. It was found that the intense peaks of HFE are located at 2950 cm^−1^ and 2856 cm^−1^, in addition to the stretching vibration peaks of -CH_3_ and -CH_2_. At 1630 cm^−1^ is the double-bond absorption peak of hydroxyethyl acrylate. The absence of double-bond peaks of HFA at 1650~1630 cm^−1^ indicates the completion of the radical polymerization reaction [29,30]. The weak absorption peak at 3465 cm^−1^ corresponds to a small amount of -OH from the HEAA. The SMA contains a lengthy CH_2_ side chain, with characteristic absorption peaks at 2857 cm^−1^ and 1440 cm^−1^. Characteristic peaks of C=O and C-O corresponding to perfluorohexyl ethyl acrylate are located at 1739 cm^−1^ and 1238 cm^−1^. The stretching vibration peaks of the C-F bond in the -CF_2_ and -CF_3_ are located at 1162 cm^−1^ and 780 cm^−1^, respectively, whereas moderately sharp peaks located at 2927 and 2856 cm^−1^ are observed for the Ag@FQS, corresponding to the C-H bond stretching vibrations of the methyl and methylene groups in long-chain quaternary ammonium groups. Broad and strong absorption peaks at 1070 cm^−1^ and 1155 cm^−1^ (s, Si-O) correspond to the presence of a large number of Si-O bonds in the Ag@FQS skeleton structure. According to reported studies [31], the FTIR spectrum of the quaternary ammonium group typically reveals a peak of medium-to-low intensity near 1470 cm^−1^. Therefore, the peak at 1472 cm^−1^ (m, C-N) could be attributed to the octadecyldimethyl quaternary ammonium group in the Ag@FQS. C-F bonds attributed to the R_f_ segments in -CF_2_- and -CF_3_ from long-carbon-chain fluoroalkyl groups are observed at 1117 cm^−1^ and 950 cm^−1^ in the FTIR spectrum of the Ag@FQS. Nevertheless, a small peak at 3381 cm^−1^ corresponds to an extremely weak Si-OH bond, indicating a marginal amount of residual Si-OH in the Ag@FQS. In addition, the peaks of Si-O bonds are observed at 1070 cm^−1^ and 1155 cm^−1^ in the FTIR spectrum of the HFA/Ag@FQS, and the peak of the quaternary ammonium group at 1470 cm^−1^ coincides with the -CH_2_ side chain peak of the SMA in the HFA [32,33]. The FTIR results confirm that the organic fluorine-modified acrylic resin HFA is successfully combined with the antibacterial component Ag@FQS.

The XPS survey spectra of the HFA, HFA/Ag-9@FQS, Ag@FQS, Ag@QS, and SiO_2_@FQS were investigated. As shown in Figure 5b, the HFA coating comprises C, O, F, and N. In contrast, the HFA/Ag-9@FQS composite coating comprises C, O, F, N, Ag, and Si, which account for 61.03%, 14.27%, 20.41%, 1.16%, 0.92%, and 2.21%, respectively. The results demonstrate that the Ag@FQS are incorporated into the HFA coating to create HFA/Ag-9@FQS. During the heating and curing process, fluorine chains migrate to the surface of the coating easily, increasing the hydrophobicity of the coating [9,11]. The peaks of Si, N, and Ag are then separated and observed in the Si2p (Figure 5c), N1s (Figure 5d), and Ag3d (Figure 5e) spectra, respectively. The Si2p (Figure 5c) peak in Ag@FQS and HFA/Ag-9@FQS is divided into two peaks which correspond to Si-O and Si-C bonds at binding energies of 103.5 eV and 102.06 eV, respectively [34]. According to the N1s analysis (Figure 5d), the peak of the quaternary ammonium N^+^ corresponds to the peak of the Ag@FQS binding energy near 401.03 eV. Only the N-C and N-H bond peaks of the secondary amino group are observed at 399.40 and 397.61 eV in the HFA, respectively [35]. Compared to the peaks observed for the Ag@FQS and HFA, the characteristic peaks of the quaternary ammonium group and the secondary amino group are observed in the HFA/Ag-9@FQS. The HFA and HFA/Ag-9@FQS exhibit a narrow Ag3d spectrum peak (Figure 5e) [36,37].

Figure 5f shows the XRD analysis of the HFA, HFA/Ag-9@FQS, Ag@FQS, Ag@QS, and SiO_2_@FQS composites. The Ag@FQS, Ag@QS, and all SiO_2_@FQS exhibit a broad dispersion peak at 2θ = 21.6° indicative of an amorphous silicon structure. The Ag@FQS and Ag@QS exhibit absorption peaks at 2θ = 38.1°, 44.2°, 64.4°, 77.4°, and 81.5° which are ascribed to the (111), (200), (220), (311), and (222) crystal planes of silver (PDF#01-087-0597) [38], respectively, indicating that AgNPs were successfully incorporated into the SiO_2_@FQS. The HFA copolymer only exhibits a broad dispersion peak at 2θ = 19.4°, which indicates of an amorphous structure [39]. Through the addition of 9% Ag@FQS nanoparticles to the polymer, the peak at 2θ = 20.1° becomes significantly weaker and shifts to a lower 2θ angle, indicating that the addition of nanoparticles increases the spatial site resistance of the polymer [40]. Additionally, a significantly higher peak is observed between 2θ = 35° and 45°, indicating that the addition of Ag@FQS alters the microstructure and morphology of the polymer.

**Figure 6:**
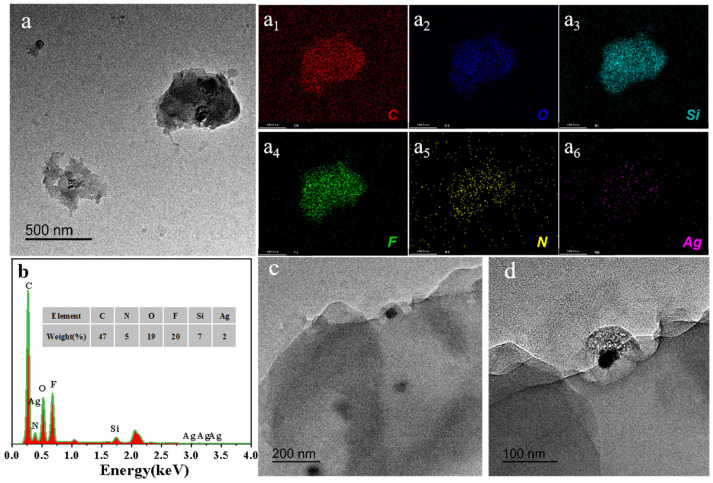
TEM image (**a**), corresponding element mapping (**a1**–**a6**), and element content (**b**) of Ag@FQS. TEM images of HFA/Ag-9@FQS (**c**,**d**).

As shown in Figure 6a(a1–a6),b, the structure contains significant amounts of evenly distributed C, O, Si, F, N, and Ag. As the additional amount of Ag@FQS is only 9%, the Ag content is low after encapsulation in the HFA for composite coating. As can be observed in Figure 6c and the corresponding enlarged high-resolution TEM image in Figure 6d, Ag@FQS are encapsulated in the HFA and exhibit strong interfacial adhesion, which is extremely advantageous for the synergistic effect of hydrophobicity, wear resistance, and antibacterial properties.

### 3.2. Surface Morphology and Wettability of HFA and HFA/Ag@FQS

**Figure 7:**
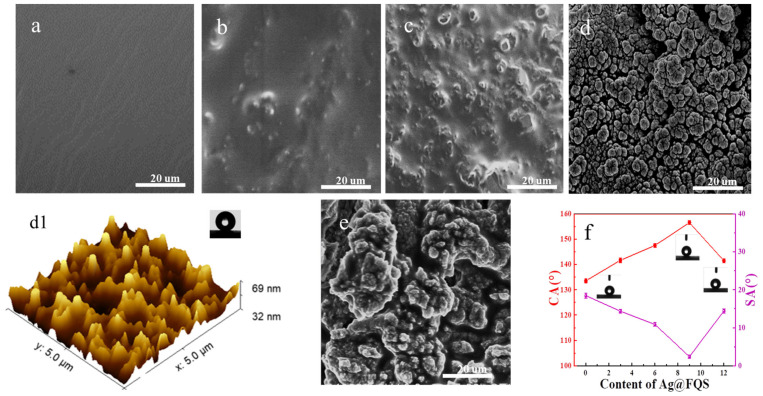
SEM images of HFA (**a**), HFA/Ag-3@FQS (**b**), HFA/Ag-6@FQS (**c**), and HFA/Ag-9@FQS (**d**), and HFA/Ag-12@FQS coatings (**e**). Three-dimensional AFM image of HFA/Ag-9@FQS coating (**d1**). Effect of Ag@FQS concentration on CA and SA of composite coating (**f**).

SEM and AFM are employed to examine the surface topographies of the HFA, HFA/Ag-3@FQS, HFA/Ag-6@FQS, HFA/Ag-9@FQS, and HFA/Ag-12@FQS composite coatings. As shown in Figure 7a, the coating surface of the HFA is relatively smooth. When the amount of Ag@FQS is increased, the surface roughness of the composite coating gradually increases (Figure 7b–e). When the amount of Ag@FQS is less than 9%, some particles are embedded in the resin matrix. When the amount of Ag@FQS reaches 9%, the nanoparticles are stacked on the coating surface to form a bumpy, rough micro–nanoscale structure, resulting a coating roughness of 6.963 nm (Figure 7d,d1). With the help of low-surface-energy fluorine, the HFA/Ag-9@FQS coating has excellent superhydrophobic property. However, an excessive amount of nanoparticles accumulate on the coating’s surface to form a micron-scale mastoid which destroys the micro–nanoscale rough structure and affects the hydrophobicity (Figure 7e).

The CA and SA of the composite coating were measured to further investigate the effect of the Ag@FQS on the hydrophobicity of the composite coating. The CA and SA of the HFA coating without the addition of Ag@FQS are 133° and 18°, respectively (Figure 7f), indicating that the prepared HFA exhibits hydrophobicity. The CA of the composite coating tends to increase and then decrease with an increase in the Ag@FQS concentration, while the SA decreases and then increases. This result is primarily related to the gradual formation of rough micro–nanoscale structures on the coating’s surface. When the Ag@FQS concentration is 9%, the HFA/Ag-9@FQS composite coating achieves a superhydrophobic state with a CA of 156° and an SA of 2°.Nonetheless, with a further increase in the Ag@FQS concentration, a large number of Ag@FQS aggregates form and destroy the rough structure of the Ag@FQS, and the hydrophobic property decreases.

### 3.3. Durability of HFA/Ag@FQS Composite Coating

**Figure 8:**
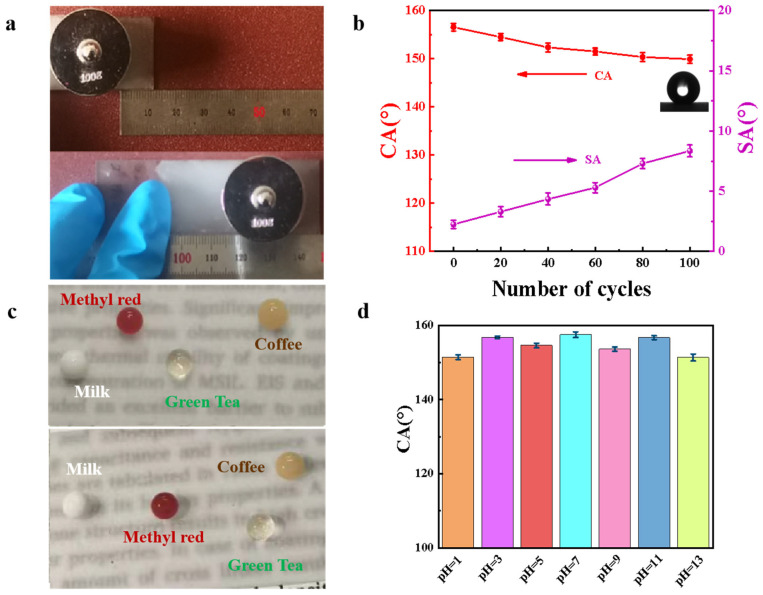
(**a**) Photo of friction test of HFA/Ag-9@FQS coatings; (**b**) change in CA and SA of superhydrophobic coatings after 0–100 cycles of friction; (**c**) hydrophobicity of different droplets on composite coatings before (**top**) and after (**bottom**) abrasion; (**d**) CA of composite coating after 60 h of immersion in solutions with different pH values.

For long-term daily use, the mechanical stability of a coating is very important. Therefore, the HFA/Ag-9@FQS superhydrophobic coating was subjected to abrasion using 240-mesh sandpaper under a load of 1 N (Figure 8a). Figure 8b demonstrates that with an increasing number of friction cycles the CA of the superhydrophobic coating decreases, while the SA increases. After 100 cycles, the CA decreased from 156° to 151°, with the SA increasing to 7°, but the coating still remained superhydrophobic. It was found that droplets of tea, dyed water, milk, and coffee remained spherical on the coating (Figure 8c, bottom), which was subjected to 100 cycles of abrasion with no scratches, showing excellent liquid repellency similar to the coating (Figure 8c, top) before abrasion. The CA of the HFA/Ag-9@FQS coating after immersion at different pH values remained greater than 150° (Figure 8d), indicating that the composite coating exhibits excellent acid and alkali resistance. These results suggest that the composite coating possesses durable superhydrophobicity [41,42].

### 3.4. Self-Cleaning Performance of HFA/Ag@FQS Coatings

**Figure 9:**
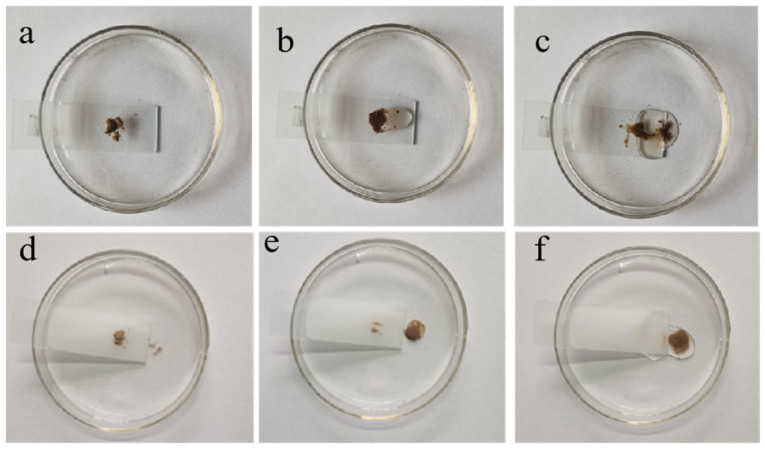
(**a**–**c**) Self-cleaning test with dirt on pristine glass; (**d**–**f**) self-cleaning test with dirt on coated glass.

In order to evaluate the self-cleaning capability of the HFA/Ag@FQS coating, soil pollutants were placed on common and coated glass sheets. As can be clearly observed from Figure 9a–c, water can easily wet the surface of the glass, and a large amount of water failed to wash the soil from the common glass sheet. It does not effectively clean the surface of the glass. In Figure 9d–f, the dust on the coating surface was easily removed with water flow, leaving a clean surface for the HFA/Ag-9@FQS coating. This is because the rough micro–nanoscale structure of the coating surface forms a kind of “air cushion” for the water droplets which makes it difficult for the water droplets to stay on the surface; therefore, the droplets can only roll down, quickly carrying away dirt, resulting in an excellent self-cleaning effect.

## 4. Antibacterial Properties of HFA/Ag@FQS Composite Coatings

**Figure 10:**
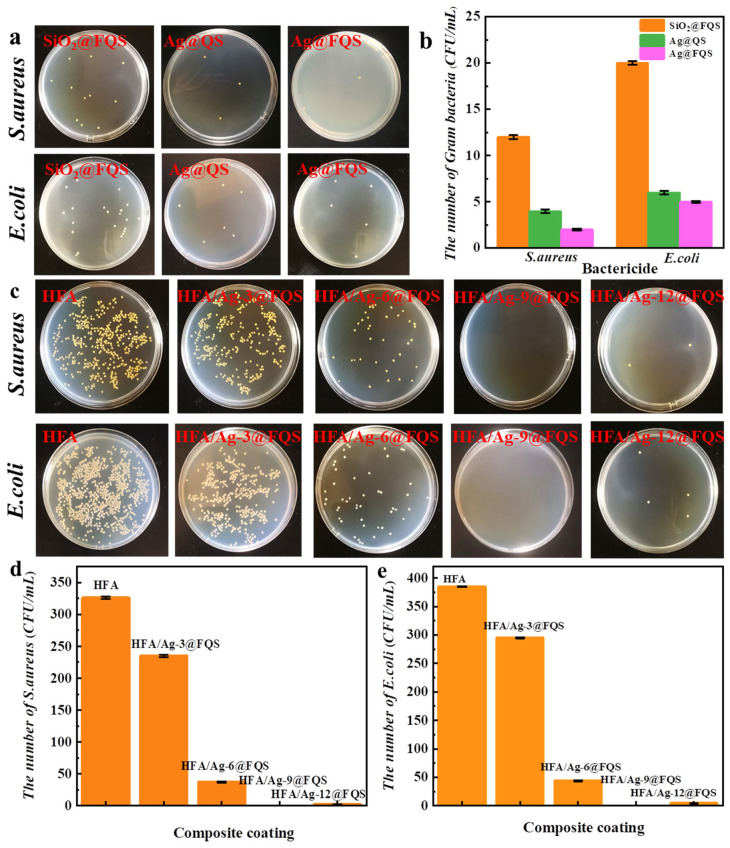
(**a**) Bactericidal properties of different bactericidal particles against *S. aureus* and *E. coli*, (**b**) statistical chart of number of viable bacteria remaining after *S. aureus* and *E. coli* extermination by different bactericidal granules, (**c**) antibacterial properties of composite coatings with different proportions of Ag@FQS against *S. aureus* and *E. coli*, (**d**) histogram of number of *S. aureus* bacteria on surfaces of composite coatings with different proportions of Ag@FQS, and (**e**) histogram of number of *E. coli* bacteria on surfaces of composite coatings with different proportions of Ag@FQS.

The plate-count method was utilized to evaluate the antibacterial activity of various bactericides (SiO_2_@FQS, Ag@QS, and Ag@FQS) and composite coatings with varying Ag@FQS content against *E. coli* and *S. aureus*, and the results are shown in Figure 10. After antimicrobial treatment, as shown in Figure 10a, the numbers of *E. coli* and *S. aureus* colonies on the agar medium (AGAR) with Ag@QS and Ag@FQS are significantly lower than those of the control SiO_2_@FQS. Figure 10b provides a quantification of the number of viable bacterial *E. coli* and *S. aureus* colonies on the agar medium (AGAR). As can be see, SiO_2_@FQS had the highest number of live E. coli and *S. aureus*, i.e., 12, 18 CFU/mL, while Ag@FQS had the lowest, i.e., 5 CFU/mL, which means that Ag@FQS has better antibacterial performance. As shown in Figure 10c, the corresponding AGAR plate for the HFA coating is covered with dense colonies of both bacteria, indicating that bacteria grow on the HFA surface. With an increase in the Ag@FQS concentration, fewer colonies of the two types of bacteria are observed on the corresponding AGAR plate. At an Ag@FQS concentration of 9%, colonies are not visible on the corresponding AGAR plate because the addition of 9% Ag@FQS to the composite coating makes it extremely hydrophobic. Meanwhile, the coating is not only resistant to bacterial adhesion but is also bactericidal. Although the bactericidal activity of the coating is enhanced with an increase in the Ag@FQS concentration to 12%, the decreased hydrophobicity of the coating leads to a slight increase in the number of colonies on the coating (Figure 10d). The numbers of viable Escherichia coli bacteria detected on the surfaces of the Ag@FQS with different proportions were 388, 293, 58, 0, and 16 CFU/mL, respectively (Figure 10e). The HFA/Ag-9@FQS composite coating showed the best antibacterial effect, which may be because the composite coating reached a superhydrophobic state with the appropriate amount of Ag@FQS and bacteria could not attach to the surface of the coating, thus improving the antibacterial activity of the coating.

**Figure 11:**
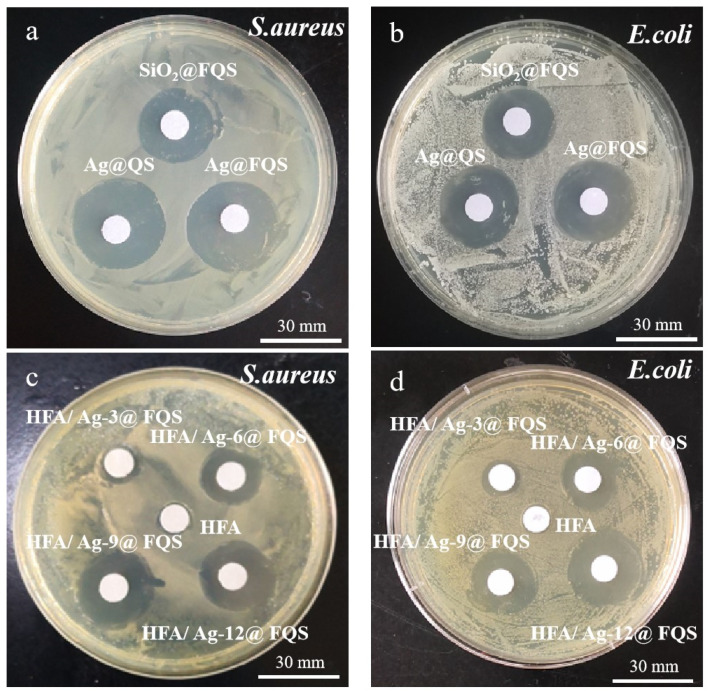
Bactericidal properties of HFA and its composite coatings HFA, HFA/Ag-3@FQS, HFA/Ag-6@FQS, HFA/Ag-9@FQS, and HFA/Ag-12@FQS, with (**a**,**c**) for *S. aureus* and (**b**,**d**) for *E. coli*.

The various antibacterial effects of SiO_2_@FQS, Ag@QS, and Ag@FQS against *S. aureus* and *E. coli* are shown in Figure 11a,b. Ag@QS and Ag@FQS exhibit comparable antibacterial effects, indicating that they exhibit the same antibacterial property, which is more potent than that of SiO_2_@FQS. It should be noted that the Ag^+^ released by AgNPs can bind with the sulfhydryl group (-SH), which is the active group of bacterial proteins, thereby inactivating the respiratory enzymes of the bacteria and killing them. However, the organic silicon quaternary ammonium in SiO_2_@FQS directly kills bacteria via electrostatic adsorption with a negative charge on the bacterial surface. As shown in Figure 11c,d, with the exception of the HFA, bacteriostatic rings are visible around other samples, and the diameter of the bacteriostatic rings increases gradually with an increase in the Ag@FQS concentration. The HFA/Ag-3@FQS samples exhibit an inhibition ring of 8.3 mm against *E. coli* and an inhibition ring of 9.2 mm against *S. aureus*. In contrast, the diameters of the bacteriostatic rings in the HFA/Ag-9@FQS sample are 27.9 mm for *E. coli* and 29 mm for *S. aureus*. The HFA/Ag@FQS inhibits *S. aureus* more effectively than *E. coli*, which is consistent with results reported previously [43]. This result is primarily attributed to the fact that the quaternary ammonium in the structure is capable of inactivating enzymes and denaturing proteins in bacterial cells. In addition, large quantities of Ag^+^ and the reactive oxygen species released by Ag particles may cause an oxidative stress reaction, thereby accelerating bacterial death.

**Figure 12:**
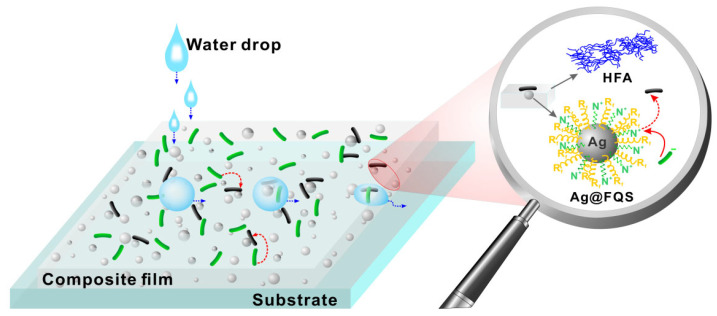
The mechanism of the antibacterial effect of the HFA/Ag-9@FQS composite coating.

As shown in Figure 12, the excellent antibacterial property of the HFA/Ag-9@FQS composite coating contributes to its antibacterial adhesion and bactericidal properties, as can be noted from the analysis above. Firstly, the HFA and Ag@FQS contain fluorine, and during the heating and curing processes, the fluorocarbon chain segments tend to accumulate on the surface to decrease the surface energy of the coating layer. Secondly, the addition of Ag@FQS enhances the surface roughness and alters the microscopic morphology of the HFA-based coating, which is mainly responsible for the superhydrophobic antibacterial adhesion of the composite containing 9% Ag@FQS. The superhydrophobic coatings greatly reduce the actual contact area between the surface of the material and the bacteria, thus resulting in the low adsorption and easy desorption of the protein, which allows bacteria to be easily removed before they form a biofilm on the surface [44,45]. Negatively charged bacteria are then killed by the quaternary ammonium in the Ag@FQS, and the corpses of the dead bacteria remain on the coating’s surface, reducing the antibacterial properties of the quaternary ammonium. Finally, free Ag^+^ from the Ag NPs in the carrier is slowly released to continuously kill bacteria. Three mechanisms contribute to the antimicrobial activity of Ag NP-based materials: oxidative stress caused by reactive oxygen species generated on the silver surface [46,47]; interactions between silver ions and -SH groups of enzymes and proteins that affect cellular respiration and damage to bacterial membranes caused by the adsorbed Ag NPs.

## 5. Conclusions

In this work, we designed a long carbon-chain hydrophobic fluorinated acrylic resin and compounded it with Ag@FQS, nanoparticles to obtain self-cleaning superhydrophobic composite coatings with antibacterial properties. The synergistic action of the quaternary ammonium salts and silver nanostructures caused the coating to show a dual antibacterial effect. The superhydrophobic self-cleaning surface can efficiently prevent the adhesion of the contaminant, which extends the service time of the coating. In addition, the coating is durable and remains superhydrophobic even after 100 abrasion cycles and 60 h of immersion in chemical solutions, demonstrating outstanding wear resistance and acid/alkali stability. The coating exhibits excellent antibacterial properties due to the synergistic effect of the quaternary ammonium N^+^ and Ag^+^ in the nanoparticles. This is expected to provide unique advantages for self-cleaning and antibacterial applications.

## Data Availability

The data presented in this study are available on request from the corresponding author.
